# Global health and the elite capture of decolonization: On reformism and the possibilities of alternate paths

**DOI:** 10.1371/journal.pgph.0002103

**Published:** 2023-06-29

**Authors:** Daniel W. Krugman

**Affiliations:** Department of International Health, Johns Hopkins Bloomberg School of Public Health, Baltimore, Maryland, United States of America; APHRC, KENYA

## Abstract

Global Health is experiencing a moment of reckoning over the field’s legacy and current structuring in a world facing multiple, intersecting challenges to health. While “decolonization” has emerged as the dominant frame to imagine change in the field, what the concept refers to and entails has become increasingly unclear. Despite warnings, the concept is now being used by elite Global North institutions and organization to imagine their reformation. In this article, I attempt to provide clarity to the issue of conceptualizing change in Global Health. By first outlining a brief history of decolonial thought and then exploring the current state of the decolonizing global health literature, I show a profound disjuncture between popularized calls for decolonization in Global Health and other theorizations of the term. I then argue that the diluting of “decolonization” into a depoliticized vision of reforming the inherently colonial and capitalistic institutions and organizations of Global Health is an example of “elite capture”—the coopting and reconfiguration of radical, liberatory theories and concepts then used by elites for their own gain. Showing how this elite capture has facilitated harm within the field and beyond, I conclude by calling for resistance to elite capture in all its forms.

## Introduction

The usage of the word “decolonization” to name and frame efforts of change in Global Health has reached a crucial juncture. Dizzyingly, over the past three-odd years a maelstrom of articles, programs, and conferences have employed the word to pinpoint issues with the field and frame solutions to them. While everything from humanitarianism to education, research partnerships, and funding has been called to be “decolonized,” what “decolonization” entails and how it happens is undefined and underdetermined. In the scholarly ecosystem of talks, articles, and other public products that engage with “decolonization” in the context of Global Health, definitions contradict, ideologies clash, and visions of change contest. Not only is the word now being widely used by actors in the field without clear definition, but also it has been increasingly adapted into the lexicon of Global Health institutions and organizations. Despite a multitude of warnings about the potential harms of using the word [1–6], “decolonization” has paradoxically become the favored word of powerful, elite global North organizations and institutions from the Johns Hopkins School of Public Health to the Bill and Melinda Gates Foundation to name the altering of their internal dynamics and their place in the broader field. Further, these entities and their peers are themselves beginning to contribute to the discourse of what “decolonization” is and how it is done [7]. In the rather short history of decolonization’s dominant rise to the top of Global Health’s agenda and glossary, contradiction and confusion have proceeded clarity and collectivity. The only clear trend seems to be while the word’s usage has dramatically proliferated amongst both individual actors and powerful organizations, what it means and evokes has become increasingly uncertain.

Unsurprisingly, critiques of “decolonization” and/or the ways it is being used have abounded. Chaudarai et al. warn against “reformist” goals and programs while arguing for movement towards decolonial thought to create a radical alterity of how global health is understood and practiced [5]. Opara calls for the “decolonization of decolonization” and grounding ideas of change in the theories and thinking of the Global Majority who gave life to the word [6]. Hirsch [1] exposes the limits of changing elite, global North academic institutions where Global Health is currently housed under the framework of decolonization. Keshri and Bhaumik [8] assert how decolonization must focus attention on the material “feudal structure of Global Health” in addition to symbolic regimes. Contractor and Dasgupta [9] argue that a decolonization of Global Health is insufficient without intersecting with anti-hierarchical movements in formerly colonized localities where elites seize power in the wake of Western withdrawal. Hindmarch and Hillier call for the “indigenizing” conceptualizations of change, and Global Health itself, in order to move away from persistent colonial ontologies that underline the word decolonization [10].

Yet, a seemingly slow, gradual advance of “decolonization” toward being a blanket word for naming and articulating social change in the field of Global Health has continued. As other scholars and activists have begun to locate [2, 10–13], the word “decolonization” has become depoliticized and decontextualized to the point where it has been rendered more meaningless than meaningful. In this, the global North powers who are traditionally the targets of decolonial critique feel comfortable using the term themselves. While this trend can simply be seen as the fashioning of “decolonization” as a “buzzword” then spread around the field, in this essay I contend that dismissing this phenomenon as such would be a crucial misstep. Rather, I argue that the “buzzwordification” of decolonization is far from uncontrollable or a “natural” progression of a word moving through a global social arena. Instead of reducing this analysis to simplistic answers, which helps obscure and evade responsibility for the consequences of this phenomenon I will soon outline, I explore *why* this depoliticization has so widely occurred and what can be revealed about social change in Global Health from that investigation.

Following these critiques of “decolonizing Global Health” from inside the field and recent works on the depoliticization of the word “decolonization” in Western academia from beyond the field [14–16], this article introduces preliminary thinking from a larger ongoing anthropological project on the use of the word “decolonization” inside Global Health. Grounded in linguistic anthropological inquiry and departing from past studies on the interpretations of the word [17, 18], I conduct a brief discourse analysis of “decolonization” and its usage inside the field of Global Health. In this anthropological exercise, I treat “decolonization” as a discursive object of study—a word with underdetermined and contested meanings, associations, and representations. By reviewing both the broader history of the word and its brief employment inside the field, I reveal some basic patterns of how “decolonization” is used inside the field of Global Health and how it relates to broader trends and social theories. In all, I begin to demystify why “decolonization” has evolved in the way that it has and interrogate the consequences of this evolution.

Due to the nature of this investigation, no patients or public were involved in the conceptualization or conduct of the study, and, thus, no ethics approval was needed or obtained.

I proceed as follows. First, I will offer an introductory history to the word decolonization and the different intellectual, social, and conceptual movements it has developed with throughout the past seven centuries. Then reviewing the brief history of “decolonization” in Global Health and using definitions, claims, and recommendations found in early and popular public works as “artifacts,” I argue that a political economy of conceptualizations has developed underneath the term “decolonization” and show how ideas, projections, and visions that define “decolonization” in relation to liberal reformist ideologies have drastically disproportionate influence on the broader field. Placing this liberal reformist dominance vis-à-vis the history of the word “decolonization” and evoking the work of philosopher Olúfémi Táíwò, I contend that Global Health is advancing the “elite capture” of decolonization. Using a recent Johns Hopkins Bloomberg School of Public Health article on how to decolonize Global Health through four individualistic steps as an example of this elite capture, I discuss the consequences of reformist pursuits and coopting radical language. Finally, I conclude that elite capture in all its forms must be resisted if true change is to be realized in Global Health.

Before continuing, I offer a brief note situating myself in relation to the ideas I am discussing and the people whose theories and ideas I am discussing. My perspectives here are inherently shaped by my positionality as a white, cisgender settler who was born, raised, and labors on land seized from the Piscataway and Susquehannock Nations that is now called “Baltimore” in the unceded settler-colonial nation-state of the United States of America. I am trained at the intersections of linguistic, cultural, and medical anthropology—a field created by and for colonial pursuits and deeply entrenched in colonial ontologies—at elite global North institutions. Thus, I approach decolonial theory as a student and its associated social movements as an accomplice. I am limited by the fact that I have not and cannot fully live and understand the struggles of colonized people. Similarly, my interpretations of theories and ontologies emerging from these struggles are inherently influenced by my specific geographic location, life history, and political education. Given this positionality, my goal in this article is not to advance decolonial theory, define what “decolonization” is, or become a leader in this movement inside or beyond Global Health. Rather, I seek to use my privilege as a white cisgender man, perspectives as a person who works inside and studies powerful Global Health spaces, and skills as a scholar trained in critical social and linguistic inquiry to critique and expose powerful forces that are restricting and undermining certain visions of change. In this, I also do not work from altruistic sentiments. I work from an explicit political position and in solidarity with all who understand that “Global Health is one of the most important global sites in which thought and action must change if the colonial order is to be unmade, reimagined, and remade” [10] and labor towards the end of the field and the world it helps to support.

## A brief history of decolonial thought

“Decolonization” is a word with a long, contested history that is much too large to be fully articulated in this space. Thus, here I will follow the history of *decolonial thought*—a school of thinking birthed by scholar-revolutionaries who theorized the concept while on the frontlines of liberation struggles in colonized regions. Before beginning, it must be clearly stated that this brief overview is in no way complete, nor is the “truth” of decolonization. Decolonial theory is in and of itself is a diverse and broad categorization of thought that is not homogenous. Not only are there other schools of thinking about anti-colonial, anti-imperial, and anti-white supremacist thought, such as *postcolonialism* [19], but also many scholars around the world including in formerly colonized region refute the idea of post-independence decolonization and decolonial thinking [20]. In other words, here I overview one milieu of thought because of its intrinsic connections to the material struggles of 20^th^ century decolonization, its continued connection to the frontlines of anti-colonial and anti-imperial movements today, and prominence outside (and to an extent inside) Global Health conversations.

Decolonial thought began the moment European settlers began conquering future colonies and native peoples thought and conversed about resisting them and reclaiming sovereignty [21, 22]. Since 1492 “decolonization” is a concept that has existed in myriad ways in different times and places, but at its core been intimately connected with the practices of preserving ways of being, fighting off settler encroachment onto these practices and knowledges, and reclaiming territorial sovereignty of conquered lands [22, 23]. The roots of decolonial thought today must be seen as a constant evolution of intertwined thought and action that has been dominantly housed in languages and mediums of knowledge creation and transfer unseen and unintelligible to the European-birthed academy.

It was during what is commonly known as the “decolonial era” beginning in the early 20^th^ century that decolonial thought started to be advanced through Romance languages and more clearly seen by the colonizing, imperializing world. While decolonial thought was actively being pursued in all colonized places, revolutionary thinkers in Africa developed particularly salient thoughts on what decolonization would entail. Amilcar Cabral, the leader of the independence struggles of Cape Verde and Guinea-Bissau, became the blueprint [24]. Writing on the formulation of national culture, the role of the masses in liberation movements, and theories of radical change, underlining his work was a central praxis: that decolonial thought is only produced as part of and through participation in liberatory struggles [25]. Frantz Fanon built upon this idea through his book *The Wretched of the Earth* [21].While fighting French occupation in Algeria, Fanon outlined how decolonization was not only the political sovereignty, but the total expulsion of European-introduced social organizations, ways of being, and economic systems to create a “new humanity” amongst formerly colonized places. Walter Rodney, a Guyanese native and Pan-Africanist freedom fighter, added the additional angle of Marxist struggle to decolonization. Intersecting Marxist theory with race and colonial relations, he argued that African nations could not truly decolonize without separating from European economic control and seizing wealth from the internal “national bourgeoisie” of these nations who supported the ongoing economic relations [26, 27].

If “decolonization” to these thinkers is the total eradication of the colonial system including capitalist economics, political organization of the nation-state, and colonial relations of power, and today we exist in a word dominantly structured by these systems [22, 28, 29], “decolonization” in this line of thought must be seen as a task that was never completed. In conversation with the works of Argentinian Marxist Enrique Dussel [30, 31], Peruvian sociologist Anibal Quijano coined *coloniality* to describe this continuation of the colonial “matrix of power” that structures knowledge and thinking into the modern day [32]. To Quijano, and many others who have helped develop the school of de/coloniality [33–35], concepts such as gender [36], being [37], development [38], and others organizing principles of modern society are largely shaped by Western Enlightenment-derived connotations of these ideas. Functionally, these ideas are designed to actively reinforce the global political economy of North Atlantic domination. With these thinkers [33], Argentinian theorist Walter Mignolo has led the development of *decoloniality* [34, 39]. In his words, *decoloniality* is “to delink…from that overall structure of knowledge to engage in an epistemic reconstitution…of ways of thinking, languages, ways of life and being in the world that the rhetoric of modernity disavowed and the logic of coloniality implement” created from taking the “decolonial option” in one’s thought and praxis [40].

While decolonial thought today must still be seen as primarily being owned and advanced by communities fighting for epistemological, political, and economic autonomy across the world, it is also being advanced within the structures and spaces of academia. Examples are plentiful and vastly interconnected, ranging from theorizing demands of Land Back [23, 41], Palestinian liberation [42], and the unified struggles between the two [43–45]. Relatedly, following Mignolo’s conceptualization of *epistemic disobedience*—an individual’s act of divorcing their thought from currently Western and colonial hegemonic epistemological structure that confines knowledge and its production [39, 46, 47]—scholars have developed deep understandings of comparative global epistemologies, the global political economy of epistemology, and breaking apart epistemological power structures is being established [48–50]. The connecting forces between these different scholars and their intellectual projects of decolonial thought are, first, their unwavering contestation that decolonial thought must be connected to anti-colonial, anti-capitalist, and anti-imperial *action*; and second, that decolonial theorization must be linked to and in conversation with the depth and history of the word inside and outside of academic spaces [16, 51–53].

However, as Tuck and Yang located in 2012, the word “decolonization” is being used to name and articulate social change critically unconnected to this scholarship and ignoring the politics of it [15]. In their now famous piece, they state plainly, “one trend we have noticed, with growing apprehension, is the ease with which the language of decolonization has been superficially adopted into education and other social sciences, supplanting prior ways of talking about social justice, critical methodologies, or approaches which decenter settler perspectives.” [15] While it is unclear when, how, and in which discipline “decolonization” began to be used as a metaphor for reformist shifts of power as Tuck and Yang locate, it is clear that it was widespread enough by the year 2012 that these two professors deeply connected with decolonial thought and Indigenous and colonials struggle could see this shift clearly. Post 2012, the trends of “settler moves to innocence” [15] using decolonization but without connection to decolonial thinking or political have continued to proliferate to unseen levels of usage in many academic disciplines [18].

## A discourse analysis of decolonizing global health

It is in this scholarly ecosystem into which the word “decolonization” entered into the field of Global Health. Given the size of the field, its diversity, and connections to other fields such as anthropology and International Studies which began debating decolonization of their respective fields as early as the 1990s, it is likely scholars in Global Health have been engaging with decolonization the word and decolonial thought the intellectual moment much longer than what is in the literature and other primary sources, and that “decolonizing” Global Health was conceptualized based on a variety of prior theorizations of the word “decolonization.” However, as others have also noted [10, 54, 55], the catalyst for the word’s entry into the field was student-led conferences directly and indirectly inspired by the 2015 #RhodesMustFall protests in South Africa and their usage of “decolonization” to bring more attention to inequalities in academia [56]. Explicit “decolonize Global Health” student-led groups and events such as ones held at Duke, Harvard, Karolinska and others beginning in early 2019 and lasting throughout pre-COVID-19 2020 were the first and most prominent places where decolonization as a paradigm for social change in Global Health was introduced.

### Mapping the literature

Thus, there is most likely no single starting place for decolonizing global health and rather a multitude of entry points the word and its different associated meanings entered into the disciplinary psyche. However, very quickly after this complex entry into the field certain ideas of what decolonizing Global Health is emerged. This can be seen in what happened after these conferences and the rapid proliferation of the term’s usage during the COVID-19 pandemic. Table 1 begins to tell this story, showing the three earliest articles directly referencing and defining “decolonizing Global Health” via a Scopus title-abstract-keyword search (“Global Health” AND “decolon*”) and limited to Global Health-focused journals. The number of article citations and, when available, the impressions provided by the journal have been included to show the basic of impact these articles have had.

**Table 1:** Earliest decolonizing global health conceptualizations.

Publish date	Journal	Title	Conceptualization of “decolonizing Global Health”	Citations	Impressions
April 9, 2019	Global Health Promotion	Climate change and Indigenous Health Promotion	“It is intrinsically linked with a process of reclaiming and centering Indigenous ways of knowing and doing. Decolonization of health promotion also requires the dismantling of institutional structures and systems that support racist practices and perpetuate inequities. This needs to be coupled with devolution of power to Indigenous communities and a commitment to uphold Indigenous peoples’ rights to self-determination.” [57]	64	N/A
March 25, 2020	BMJ Global Health	The promise and pitfalls of social science research in an emergency: lessons from studying the Zika epidemic in Brazil, 2015–2016	“However, important questions remain as to how this research should be conducted in a way that is ethical, practical, appropriate and of high quality. These questions relate strongly to debates around the decolonisation of global health, which is the attempt to address the entrenched power asymmetries in global health partnerships in the conduct of research.” [58]	10	20
September 2, 2020	International Health	Decolonising global health: transnational research partnerships under the spotlight	“We refrain here from offering a normative or static definition of what decolonising global health means and accept, following Tuck and Yang, that real decolonisation needs to take place outside academia and needs to be led and abide by the principles of indigenous communities.” [54]	51	N/A

Interesting insights emerge from the earliest three articles found in this search. The first article published in a Global Health journal that defines “decolonizing Global Health” is written by a Māori scholar. Further, Jones’s definition of decolonization is that of dismantlement and abolition to allow for Indigenous perspectives and knowledge to thrive [57]. However, the following two articles begin to depart from this thinking. While the third article follows Tuck and Yang’s call on the needed materiality of decolonization in response to academics using the concept as a metaphor [15] and assert “real decolonization” happens outside the academy, they then outline the decolonization of different aspects of Global Health aspects such as transnational-research partnerships, authorship, expertise, and infrastructure [54]. That is, they call for the reform of Global Health to make it more inclusive and equitable. Similarly, the second article focuses on authorship and international connections between Global Health scholars, and declared power asymmetries in Global Health partnerships must be rectified [58].

These first articles in the decolonizing Global Health literature basis reflect the nature of the word in academic spaces and its myriad entry into the field. Rather than a singular conceptualization being used as the basis for definition, there is a disconnect between the first article, the third, and, especially, the second. While the first seems to directly draw from and be in deep conversation with decolonial theory and Indigenous praxis to theorize dismantlement of global health promotion structures and the systems that support it, the second and third seem to take a different perspective. They focus on decolonization as changing who is in the discipline, the relationships between those people, and how the structure of the discipline creates certain injustices or disadvantages. The overall structuring of the field, what it collectively seeks to do in the world, and how the field is connected to larger systems are left unaddressed.

Following the third article in particular, a wave of scholarship began focusing on the decolonization of different aspects of Global Health or the field in totality. Büyüm et al. [59] offer the broad overview of “decolonizing Global Health,” stating, “decolonizing global health advances an agenda of repoliticising and rehistoricising health through a paradigm shift, a leadership shift and a knowledge shift.” Eichbaum et al. set the agenda for education, calling for reforms such as “developing global health curricula, learning objectives, and competencies” and “equalizing access and opportunity of educational experiences” [60]. Following this lead, an extensive sub-literature on the topic has developed [61–66]. By early 2021, broad strategies and plans for field-wide “decolonization” was being offered. The “roadmap from rhetoric to reform” written by Khan et al. that outlines a three-step plan “to drive reforms” has become one of the most widely cited works in decolonizing Global Health literature [67]. Similar large, sweeping calls and theorizations attempting to answer how does decolonization happen have followed over the past two years [68–70], while other theorizations to decolonize Global Health research broadly [71], humanitarian aid [72], and funding structures [73].

At the same time, though, other scholars took a different route. Directly linking conceptualizations of “decolonization” to decolonial theory and other social theory from beyond Global Health, scholars have sought to theorize the “decolonization” of Global Health as altering Global Health’s place in the *broader* world system instead of focusing on partnerships, funding, and power asymmetries *within* the field. Early in the decolonizing Global Health conversations, Affun-Adegbulu and Adegbulu argue that “decolonization” requires “ontological pluralism in the concept of humanity” that must be enacted through the dismantlement of power structures that restrict those otherwise conceptualizations from thriving [74]. In direct response to Khan et al.’s “roadmap to reform” [67], Chaudhuri et al. pinpoint the shortcomings of these reforms and instead call for more radical dismantlement of the field’s structures and development of decolonial thought in the field [5]. Similarly, Hindmarch and Hiller directly draw from decolonial theory and contemporary conceptualizations of “decolonization” as political action and cognitive reconfiguration [10]. Using four Indigenous ontological structures to fill the holes in naturalized Western theoretical models commonly employed by Global Health actors, they declare that any turn away from dominant modes of thought necessarily requires political initiative and imperative [10]. This attention to de/coloniality is also articulated by other scholars over the past two years in the form of other broad calls to develop decolonial thought in the field [75] and in the realm of education [76, 77].

Relatedly, Abimbola articulates how “the foreign gaze” permeates across the field of Global Health [78]. Building on this, he and Bhakuni explore “epistemic injustice” inside the field in which “structurally marginalised groups are prejudicially denied interpretive resources to make sense of the world or their perception of the world” [79]. In these two works, the word “decolonization” is not used to name a process of social change in Global Health. Rather, attention is placed on how history and the global system Global Health is an integral part of creates epistemic superiority of certain modes of thought and the consequences of these processes. Similarly, Naidu [80], explicitly following Quijano [32] and other scholars of ‘epistimologies of the south’ [81], argues that *epistemic violence*, in which knowledge and expertise of local scholars and healers are displaced, disavowed, and silenced in favor of having to systematically adapt Western-colonial knowledges to be recognized, respected, and survive in the academic economy is an inherent requirement of the field. Instead of advancing “Northern ventriloquism” in which LMIC scholars “enunciate HIC ideas to access globally competitive grants and publish in high-impact journals,” Nadiu calls for “epistemic disobedience” in the tradition of Mignolo and the school of de/coloniality [80].

### Distribution of influence

Over the past three-odd years, how “decolonization” in Global Health is understood and the meanings that have become attached to it are messy and contested. As seen, and as others have also noted [5, 6, 10], two milieus of thought seemingly emerge. First, there are conceptualizations of decolonization attached to notions and ideas of *reforming* the field or certain aspects of it. Seen in the first half of the proceeding overview, these articles use “decolonization” to name programs and efforts attempting to alter relationships, practices, and structuring *inside* the field. Second, as seen in the latter half, there are conceptualizations of decolonization that look beyond the internal relations of the field and instead towards altering the epistemological and material structuring of the field. A similar distinction has been made by Shawar et al. in studying the other common Global Health discourses ‘resilience,’ ‘self-reliance,’ and ‘increasing country voice’ [82]. In their review, they find that two contesting ideologies—“reformism” operating from “neoliberal and liberal democratic ideologies” and “transformationalism” following “threads of neo-Marxist ideology”—underpin definitions and understandings of these words [82]. As they state, “these ideologies shape differences in how actors define the problem, its solutions and attribute responsibility, resulting in nuanced differences among global health actors in their understanding of resilience, self-reliance and increasing country voice” [82].

However, what is most important here is not the potential existence of two schools of thought. Not only does this need more salient research to be fully articulated, but also many of the articles in this literature seems to refuse this binary of “reformism” and “transformationalist.” A number of articles in the first part of the first section engage with radical theory while a few articles in the second section also offer reform. Rather, what is vital to explore is how these different interpretations of what “decolonization” have functioned in the broader Global Health ecosystem over the past three years. Table 2 begins to demonstrate this by showing the conceptualizations of “decolonizing Global Health” drawn from the most cited articles as of February 2023 in the same search as earlier.

**Table 2:** Most cited and circulated conceptualizations of decolonizing the field of Global Health.

Publish Date	Journal	Title	Definition	Citations	Impressions
August 5, 2020	BMJ Global Health	Decolonizing global health: If not now, when?	“Global health needs integrated, decolonised approaches advanced by individuals and institutions that address the complex interdependence between histories of imperialism with health, economic development, governance and human rights.” [59]	225	410
November 21, 2020	The Lancet	Will global health survive its decolonization?	“To decolonise global health is to remove all forms of supremacy within all spaces of global health practice, within countries, between countries, and at the global level. Supremacy is not restricted to White supremacy or male domination. It concerns what happens not only between people from HICs and LMICs but also what happens between groups and individuals within HICs and within LMICs. Supremacy is there, glaringly, in how global health organisations operate, who runs them, where they are located, who holds the purse strings, who sets the agenda, and whose views, histories, and knowledge are taken seriously. Supremacy is seen in persisting disregard for local and Indigenous knowledge, pretence of knowledge, refusal to learn from places and people too often deemed ‘inferior’, and failure to see that there are many ways of being and doing. Supremacy is there in persisting colonial and imperialist (European and otherwise) attitudes, in stark and disguised racism, White supremacy, White saviourism, and displays of class, caste, religious, and ethnic superiority, in the acquiescing tolerance for extractive capitalism, patriarchy, and much more.” [3]	169	1,203
March 23, 2021	BMJ Global Health	Decolonizing Global Health in 2021: a roadmap from rhetoric to reform	“We see ‘decolonising global health’ as a movement that fights against ingrained systems of dominance and power in the work to improve the health of populations, whether this occurs between countries, including between previously colonising and plundered nations, and within countries.” [67]	108	195

As seen, the most popular articles conceptualizing decolonization in the context of Global Health focus on the *internal* dynamics of field. Only the second article alludes changing the systems in which Global Health exists in addition to Global Health itself [3]. While the second article also ponders if Global Health would survive decolonization and connotations of dismantlement, the other two focus almost solely on the reform of Global Health organizations, people, distribution, and relationships as the horizons of decolonization. A similar trend emerges when widely circulated ideas of *how* to “decolonize global health” in the literature are examined. Table 3 show three of the most popular articles that includes recommendations on how to “decolonize” found in the search. “Popular,” here, refers to the total combined number of citations and impressions provided by the journal.

**Table 3:** Popular recommendations or steps for decolonizing global health or specific facets of it.

Year	Journal	Title	Recommendations/Suggestion	Citations	Impressions
August 5, 2020	BMJ Global Health	Decolonizing global health: If not now, when?	Paradigm shift: Repoliticise global health by grounding it in a health justice framework that acknowledges how colonialism, racism, sexism, capitalism and other harmful ‘-isms’ pose the largest threat to health equity.Leadership shift: Leadership at global agenda-setting institutions does not reflect the diversity of people these institutions are intended to serve. First, the ‘Global North’ needs to ‘lean out’ on an individual, national and institutional level to stop reproducing racist and colonialist ideologies. Knowledge shift: To avoid perpetuating the kind of racist and colonialist pandemic response we see with COVID-19, it is vital to ensure knowledge flow is not unidirectional, but instead reciprocal with contributions from the ‘Global South’ driving discussions and practice, both locally and globally; a twofold knowledge shift [59].	225	410
March, 2021	Academic Medicine	Decolonizing Global Health Education: Rethinking Institutional Partnerships and Approaches	1. Decolonizing by emphasizing patient safety 2. Decolonizing by applying fair trade principles to educational programs 3. Decolonizing by developing global health curricula, learning objectives, and competencies 4. Decolonizing by addressing power dynamics and development narratives 5. Decolonizing by equalizing access and opportunity of educational experiences [60]	132	201
March 23, 2021	BMJ Global Health	Decolonizing Global Health in 2021: a roadmap from rhetoric to reform	Step 1: identify specific ways in which organisations active in global health play interlinked roles in perpetuating inequity Step 2: publish a clear list of reforms required to decolonise global health practice, so that organisations that are committed to moving beyond statements can better respond to the decolonisation agenda in a more proactive and coordinated way. Step 3: linked to the reforms identified, develop metrics to track the progress of organisations active in global health and transparently share findings via different public channels [67].	108	195

Again, these ideas focus on making the internal structures of Global Health more equitable and less asymmetrical. From considerations for individuals and funders to change their behaviors [48] to directly calling for a clear list of reforms of the field [67] or consideration for training future professionals [60], “decolonization” is done through working through established avenues of change, through legitimized tools, and within to established organizations and the wider system. While these searches are not done scientifically, the metrics used to assess impact and popularity are imperfect, and the plethora of spoken presentations about decolonizing Global Health are not included, this rudimentary data begins to reveal what “decolonization” is and how it is done is the reformation of Global Health.

### The political economy of conceptualizing decolonization

As this small glimpse into the “decolonizing Global Health” ecosystem begins to show, the word “decolonization” inside the social arena of Global Health is a complex semantic landscape. There are a multitude of contesting and contradictory definitions and thinking attached to word being circulated around the field. However, these connotations and conceptualizations exist within a *political economy of conceptualizing decolonization* in Global Health. That is, certain notions of what “decolonization” is and how it is done are disproportionately circulated, cited, drawn from, and built upon. While calling for a diverse array of reforms to many parts of the field, dominantly, as Oti and Ncayiyana articulate, by scholars residing in global North institutions [83], these most popular works of “decolonizing Global Health” seem to be the ones that use the word decolonization to name and frame reforms of a field structurally supportive of and supported by neoliberal capitalism [84–87], complicit in imperial relations of power [88, 89], and built on and committed to coloniality [90]. In other words, and as others have begun to locate [5, 6, 10], the connecting thread behind how “decolonization” is dominantly being theorized is thinking based on and attached to a *politics of liberal reformism*. Liberal, here, refers to ideas of freedom, democracy, and societal development forged during the European Enlightenment that were exported around the world through the processes of colonization and imperialism. Reformism refers to the theory of change linked to Enlightenment thinking that social organizations, structures, and systems change the most effectively through the formalized means recognized and legitimized by those social organizations, structures, and systems. Liberal reformism, then, is the idea that change is created through reformation or slight altering of (now global) structures and systems guided by Western-derived conceptualizations of freedom, democracy, and developmentalism [38, 91]. Finally, a politic can be seen as the logics, assumptions, and ideologies with which one or a social body approaches a social issue.

A politics of reformism in Global Health is approaching the issues derived from the field’s location in capitalism and imperialism with the intentions of the altering of an implicitly colonial superstructure so that there is equality between different peoples inside that structure while those underlying forces of capitalism and Western dominance remain intact. Change framed as decolonization is seen as something that must be advocated for to the powerful, fought for in boardrooms, and pushed for inside policy arenas. While the evidence presented here is foundational and preliminary, the disproportionate power of reformist ideas of change in defining “decolonization.” should not be seen as a radical assertion. The vast majority of formalized “decolonizing Global Health” initiatives and programs are committed to advancing reform of the field or elite institutions dominantly located in wealthy settler states. On a deeper level, the basis of Global Health is a liberal vision of change. It is based on the assumptions of Western developmentism. It is built to “better the world” from within the bounds of the system. Liberal reformism is the baseline, the “normal” standpoint, the easiest and most accepted way to imagine change.

This is not to say that these definitions and recommendations produced inside the intellectual ecosystem of Global Health are incorrect or useless, nor that the reformist connotation of decolonization have become hegemonic in the field. The strategies provided as examples are essential for short-term harm reduction inside the structures that exist and can possibly create the conditions for new healing and prevention practices connected to local knowledge to emerge. It is to say, though, that the most prominent theorizations and popular recommendations that have been attached to the word “decolonization” do not consider the field’s active role in broader systems of global racial capitalism, do not seek to resist it, or are in deep conversation with decolonial theory beyond the field. Although there are a plethora of different ideas about what decolonizing Global Health is, many of which are directly linked to and are in conversation with decolonial thought and social movements, what has dominantly come to be accepted as “decolonization” is various notions of change that largely keep Global Health and the broader world system it exists inside intact.

## Elite capture and its consequences

While it is unclear specifically how “decolonization” became dominantly connected to a politics of liberal reformism, it is clear that the “decolonizing global health” movement has arrived as a small part of a larger pattern in academia where scholars in settler states are using the term to describe change inside their respective fields. As Kieron Turner notes, “higher education institutions [are] deploy[ing] ‘decolonising’ interchangeably with the liberal notion of ‘diversifying’, emptying the historical context and conceptual meanings of decolonization as rooted in the material struggles for liberation against European imperialism” [16]. It is not only likely that many of the original conceptualizations of “decolonizing Global Health” were created in relation to definitions of “decolonization” already far separated from decolonial theory and action within the academy, but of the original theorizations delivered in talks and papers what subsequently became popular largely became conceptualizations that were the most morally acceptable, understandable, and familiar to the audience it was being presented to—who are dominantly scholars, scientists, and medical providers in the Global North. Over the past three years, a scholarly ecosystem has now been formulated in which Global Health scholars writing about decolonization or actors seeking to create decolonizing programs can cite and draw from only other works on “decolonizing Global Health” from inside that ecosystem, many of which, as outlined, are not linked to broader political theory or social movement. This ecosystem now seems to be self-sustaining and continuing to grow at a breakneck pace.

This process of delinking the word decolonization from the revolutionary liberation of formerly colonized places from European-American political and economic domination outlined at the beginning of this piece to become calls for reform of an inherently colonial social organization is a clear example of what Olúfémi Táíwò has coined “elite capture” [92]. In his book of the same name, Táíwò explores how the concept of “identity politics,” which was originally introduced by the radical Black feminist Combahee River Collective in the United States as a liberatory framework for social movement, has become a mainstream talking point in American politics that looks nothing like the original concept. Mapping how identity politics was adapted and used by academics, industry leaders, and politicians, Táíwò argues that political, social, and economic elites have taken the concept with radical and liberatory potential and refashioned it for their own gain [92]. To Táíwò, this “elite capture” is a fundamental strategy of bourgeoisie power maintenance and a process that can happen with other ideas. Through radical ideologies and concepts being taken up by elites, depoliticized through using them in ways they were not intended to be, and deploying them for their own benefit, elite capture both hampers political movement and strengthens the status quo [92].

Like identity politics, the same process is unfolding inside Global Health, and academia more broadly, surrounding the concept of “decolonization.” A word that has a long, deep political and theoretical history once reserved for use and thinking inside of anti-colonial and anti-imperial social movements has been largely transformed to now describe the slight restructuring of Global North corporations, institutions, philanthropies, governments, and the superstructures they interact inside. This process is fueled by the words continuous use and circulation in academic, NGO, government, corporate, and allied spaces by people very familiar with these spaces to make sense of how the word could fit into changing these spaces. However, use inside these spaces—and among increasingly powerful people—without consulting history, theory, or the different people who originally created the idea has led to its separation from a politics of liberation as it was designed to mean. While this capture is paved with good intentions, the result is a political economy of meaning in which the word is dominantly employed to describe the reformist pursuit of equity inside the established structures of Global Health. The consequences of this process are not neutral.

Take, for example, an article published and promoted widely by the Johns Hopkins School of Public Health. In this institution-sponsored publication, colonialism is presented in the past tense, a historical event that happened and now “remnant” dynamics remain [7]. The article claims through four steps Global North practitioners and scientists can take—“asking local researchers what they think,” “changing the teaching” towards cultural competence, building local power through helping Global South medical providers with equipment, and learning an individual is a “colonizer” or “decolonizer” through an online quiz—“lingering perspectives” in the field can be “overturned” [7].

Clearly, the most “elite” school of public health in the world with over a hundred Global South and Indigenous partner organizations and thousands of followers on different social media platforms, presented “decolonization” as an individualistic act of reforming how Global Health researchers and students see themselves and interact with research partners across the world. These ideas didn’t simply arise from nothing. They are directly born from the scholarly ecosystem on changing the field that is dominated by reformist theorizations of “decolonialism” and rhetorics of reform. The scholarship on “decolonizing Global Health” has become entrenched in a reformist theory of change to the point that one of the largest mouthpieces of Global Health, which is also directly linked to corporate and state powers while having a rather long history of malpractice both in Baltimore and around the world [93], now feels comfortable using the term to conceptualize change.

A public pandering of reformist decolonization by an elite institution obviously has consequences. First, the broader movement to change power relations between colonizing forces and colonized peoples is diluted, depoliticized, and decentered to then work for the interests of power holders. When Johns Hopkins and similar wealthy and powerful Global North organizations broadcast reformist decolonization on their disproportionally influential platforms, the public’s perception of what decolonization is can be altered. These larger, more influential, and institutionalized platforms take space from more radical definitions and projects, complicating their work of real material struggles over land and resources against colonial forces. The impact this has in the boardrooms and strategy meetings of Global Health organizations from the WHO to smaller NGOs in the Global North is probably not negligible, and definitely worth critical investigation.

At the individual level, the effects of this elite capture become even more profound. Western colonization and imperialism are ongoing genocides. Many colonized people do not have a choice to be anything but decolonial in their praxis and existence. Colonial forces use the name of their struggle to push agendas that keep or expand their own power has, for me, a cisgender-white male from the colonizing class, unimaginable consequences. It is not my place to describe or contemplate this pain. The reaction to the Johns Hopkins article on different social media platforms, as well as the thoughts published by Indigenous scholars in its aftermath [94], speaks for itself.

The process of depoliticizing decolonization in Global Health, or, the afterlives of scholarship on radical concepts with reformist politics, does real harm to people and movements. Words, how they are used, and how meaning becomes attached to them, matter.

## The possibilities of alternate paths

The efforts to change Global Health and the imaginative work it takes to conceptualize that change are clearly perilous. Contrary to the original theorizations of decolonization, in just roughly three years since the word reached the mainstream of the field the dominant connotations and definitions of “decolonization” are that of liberal reformism. While the process of elite capture of the word and idea “decolonization” began long before the word was introduced to Global Health, its continued depoliticization and conceptual separation from anti-colonial and anti-imperial social movements is actively advanced by Global Health actors. While this is not done from a place of malice or ill-will by those using the term in this way, it is a choice. To actively use the word in ways that are disconnected from the people and movements it was created by, to claim that its multiple meanings are a pass to use it in uncritical ways, or to employ it to name projects of elite institutions, imperial governments, and bourgeoisie philanthropy is to advance this elite capture. The unfortunate reality for Global Health is that “decolonization” has been fashioned to largely mean the reformation of our inherently colonial, capitalist supporting institutions and organizations, which will not quell the violence that the continued colonial global political economy and capitalism continue to structure around the world.

The mission to change Global Health is at a conceptual crossroad. On one hand, the choice to use the word in ways that are divorced from history, theory, and social movements could continue to be made. There is a chance that could lead to the reforms that many desire. However, there is also a chance that the word is used until it falls out of favor and is replaced by another word to name and frame change in the field. In both scenarios, continuous harm to the people and movements who use this word in sacred and generative ways continues. On the other, a collective reorientation could be pursued. Growing from the seeds already planted in the literature that theorize the decolonization of Global Health in the light of the word’s original theorizations and political commitments, these theorizations committed to abolition and epistemological pluralversity would be grown and acted upon. Would this kind of reorientation be possible? Is the word “decolonization” doomed to suffer a conceptual death, and instead a new framework for change be pursued? I believe the answers to these questions are not for one to determine alone and instead should be thought through collectively in solidarity with both people inside of the field and those working for global social change beyond its boundaries. However, I offer three assertions and a few thoughts based on the thinking here that could help the field begin to answer these questions and move towards the latter of the two options.

First, and most immediately, collective solidarity with and connection to anti-capitalist, anti-imperialist, and anti-colonial social movements around the world must be fostered. Like conceptualizations of decolonizing Global Health connected to decolonial thought, the seeds of this already exist in many individuals’ who associate with Global Health relationships and political commitments. Given that the field has both recently and historically been understood by many of these groups around the world as part of imperialism and capitalist expansion [95], this will be a difficult task.

Second, if the scholarly ecosystem of Global Health is largely divorced from the broader literature of decolonization—and most critical political theory for that matter—it is vital that collective pursuits of change are united with what scholars beyond our field are theorizing and how they are acting. The literature of Global Health is not capable of fully educating ourselves on social change, global political theory, or radical terminology. Instead, more reading beyond the field’s corpus must be done and in writing theorizations, recommendations, musings, and work must be connected to those who have been and are at the front lines of thinking through these issues in more holistic ways. Any writing or program creation that uses radical language must be fully versed in the terms, definitions, and theories that are being used.

Finally, elite capture in all its forms must be rejected and resisted. The powers of Global Health—that being elite institutions, billionaire-funded organizations, colonizing and imperial nation-state aid structures, and the people who run them—cannot be allowed to continue diluting and depoliticizing radical language created to resist the systems they are an integral part of. This is vital for protecting the clarity of language and preserving our respectability as we attempt to work with broader social movements resisting settler-state and capitalistic violence.

Elite capture is a process that has thus far defined the usage of the word “decolonization” in Global Health. However, it is exactly that: an ongoing social process. The elite capture of decolonization is neither a natural nor finalized phenomenon. In the same way that the word’s depoliticization is fueled by myriad and uncountable small and large choices being made over and over again until the larger patterns can be seen, it can be repoliticized in the same way. Alternate possibilities of how change in Global Health is imagined and pursued exist both inside of the word decolonization and beyond it in growing connections to Black [96, 97], and Marxist [98] thought. The question is, are we bold enough to make change framed around a politics of liberation the norm?
